# Regulation of Chemokine Function: The Roles of GAG-Binding and Post-Translational Nitration

**DOI:** 10.3390/ijms18081692

**Published:** 2017-08-03

**Authors:** Sarah Thompson, Beatriz Martínez-Burgo, Krishna Mohan Sepuru, Krishna Rajarathnam, John A. Kirby, Neil S. Sheerin, Simi Ali

**Affiliations:** 1 Applied Immunobiology and Transplantation Group, Institute of Cellular Medicine, Medical School, University of Newcastle upon Tyne, Newcastle upon Tyne NE2 4HH, UK; s.thompson3@ncl.ac.uk (S.T.); b.martinez.burgo@ncl.ac.uk (B.M.-B.); john.kirby@ncl.ac.uk (J.A.K.); neil.sheerin@ncl.ac.uk (N.S.S.); 2 Department of Biochemistry and Molecular Biology, The University of Texas Medical Branch, 301 University Boulevard, Galveston, TX 77555, USA; kmsepuru@utmb.edu (K.M.S.); krrajara@utmb.edu (K.R.)

**Keywords:** chemokine-GAG interaction, synthetic peptide chemistry, PTM, chemokine nitration

## Abstract

The primary function of chemokines is to direct the migration of leukocytes to the site of injury during inflammation. The effects of chemokines are modulated by several means, including binding to G-protein coupled receptors (GPCRs), binding to glycosaminoglycans (GAGs), and through post-translational modifications (PTMs). GAGs, present on cell surfaces, bind chemokines released in response to injury. Chemokines bind leukocytes via their GPCRs, which directs migration and contributes to local inflammation. Studies have shown that GAGs or GAG-binding peptides can be used to interfere with chemokine binding and reduce leukocyte recruitment. Post-translational modifications of chemokines, such as nitration, which occurs due to the production of reactive species during oxidative stress, can also alter their biological activity. This review describes the regulation of chemokine function by GAG-binding ability and by post-translational nitration. These are both aspects of chemokine biology that could be targeted if the therapeutic potential of chemokines, like CXCL8, to modulate inflammation is to be realised.

## 1. Introduction

Chemokines are small cytokines (8–17 kDa) with chemoattractant properties that are involved in processes ranging from homeostasis to development and tissue repair. They also play essential roles in pathological conditions such as tumorigenesis, cancer metastasis and inflammatory or autoimmune disorders where they mediate the migration of leukocytes to the site of injury [1,2,3,4]. Chemokine biology also plays a role in generating immune tolerance [5]. Chemokines are classified into four subfamilies; C, CC, CXC and CX3C in relation to the location/spacing of cysteine residues within the N-terminal region.

**Figure 1:**
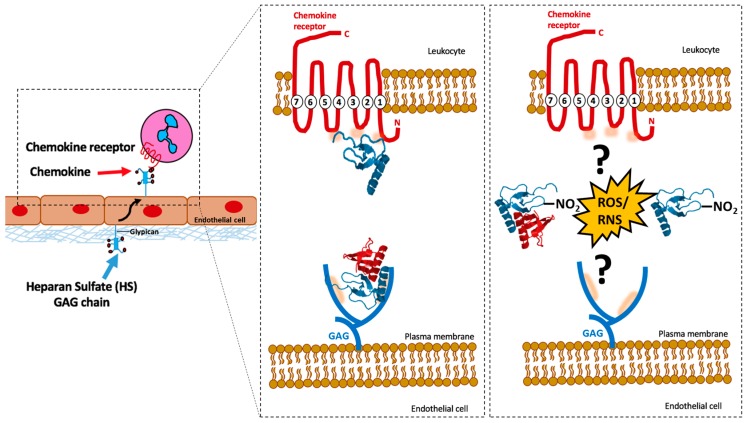
Chemokine interactions with G-protein coupled receptors (GPCRs) and glycosaminoglycans (GAGs). Chemokines bind to GAGs present on the surface of endothelial cells in a dynamic manner, creating a localised chemokine gradient and facilitating the recruitment of leukocytes. Leukocyte recruitment is a multistep process in which leukocytes tether to, roll along, and adhere to the endothelium before transmigrating out of the blood vessels. On the right, magnified image indicating specific chemokine regions involved in GPCR/GAG binding (shaded in orange), and potential consequences of stress (i.e., production of reactive oxygen species/reactive nitrogen species (ROS/RNS respectively)) on regulation of chemokine function. CXCL8 is used as an example chemokine, with the monomer shown in blue and the dimer depicted with one monomer in blue and the other in red.

The migration of immune cells is mediated through the formation of dynamic chemokine gradients, which are achieved by the binding of chemokines on glycosaminoglycans (GAGs) present on the surface of endothelial cells and in the extracellular matrix [6]. This creates an equilibrium of free and bound monomer and dimer in the proximity of the injury, resulting in haptotactic and chemotactic gradients. This allows directed movement of leukocytes from circulation to the site of injury via chemokine signalling through the G-protein coupled receptors (GPCR) [7,8]. One of many possible GAG-chemokine-receptor interaction scenarios is shown diagrammatically in Figure 1 below.

Regulation of chemokine function is essential in order to prevent excessive inflammation and allow healing after injury. This regulation can occur at many levels and can involve different aspects of chemokine biology, including epigenetic modifications which can affect chemokine production [9], the concentration and oligomeric state of the chemokine (monomer/dimer), the steepness of the chemokine gradient [10,11], the ability of the chemokine to interact with GPCRs and GAGs [7,12], and receptor signalling bias [13,14]. Post-translational modifications (PTMs) such as nitration, glycosylation, phosphorylation, and citrullination also play a critical regulatory role on chemokine function.

In this review, we will describe how chemokine function can be regulated by GAG-binding and post-translational nitration, primarily focusing on CXCL8 as a model CXC chemokine.

## 2. Chemokine and Chemokine Receptor Interactions

Chemokine receptors all share a similar structure; an extracellular N-terminal domain, seven transmembrane-spanning segments, three extracellular loops, three cytoplasmic loops and a C-terminal segment [15]. Binding of chemokine ligands to their receptors initiates a signalling cascade involving the influx of calcium, which ultimately leads to chemotaxis [7].

Targeting the interaction between chemokines and their receptors is one potential method to regulate the recruitment of leukocytes and modulate inflammation. However, this is limited by the high level of promiscuity displayed by chemokines and their receptors [16]. While some receptor-ligand interactions are specific e.g., CX3CL1-CX3CR1 or CCL20-CCR6 [15], chemokines can often bind multiple receptors, and receptors may in turn be activated by many chemokines, making it difficult to achieve a selective and specific effect when targeting these interactions [17,18]. For example, whereas CXCR1 binds CXCL8 with high affinity and CXCL6 with lower affinity, CXCR2 binds CXCL1/2/3/5/6/7/8 with high affinity [15,19,20]. In addition, there are atypical receptors (ACKR) such as ACKR1/D6 or ACKR2/DARC, that bind chemokines but do not induce G-protein signalling [21]. They act as chemokine scavengers and are thought to be involved in the regulation of the immune response. For instance, DARC present on erythrocytes is known to induce clearance of circulating CXCL8, affecting the chemokine’s ability to stimulate neutrophil recruitment [22], hence having a significant role limiting the inflammatory response.

## 3. Chemokines and GAG Interactions

**Figure 2:**
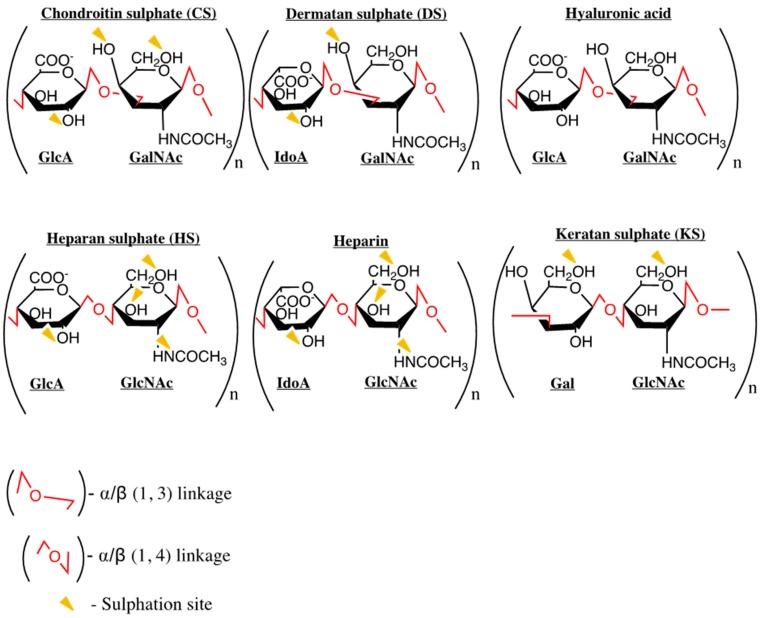
Structure and composition of GAGs. Linkages are shown in red, and sites of sulphation indicated by yellow triangles. The backbone is made up of repeating disaccharide blocks composed of uronic acid (glucuronic acid (GlcA) or iduronic acid (IdoA)), or galactose (Gal) and an amino sugar (*N*-acetyl-galactosamine (GalNAc) or *N*-acetyl-glucosamine (GlcNAc)).

GAGs such as heparan sulphate (HS), are long linear polysaccharides consisting of a repeating disaccharide unit [23] frequently covalently attached to a core protein forming proteoglycans. The main classes of proteoglycans are defined according to their distribution, homologies, and function. Common examples of HS proteoglycans are glypican, syndecan and perlecan. GAGs display varying patterns of sulphation, which in addition to carboxyl groups, confer a negative charge which is a critical determinant of chemokine binding [24]. GAGs are located primarily on the surface of endothelial cells, as macromolecular complexes with matrix proteins in the extracellular matrix (ECM), and are also secreted/shed during active inflammation [25]. They can be divided into four groups: heparin/heparan sulphate, chondroitin sulphate/dermatan sulphate, keratan sulphate, and hyaluronic acid (a non-sulphated GAG, non-covalently attached to proteins) shown in Figure 2.

Although chemokines are promiscuous to a degree in terms of receptor binding, data on GAG binding is beginning to show that chemokines interact with GAGs differently, and must be studied individually [26,27,28]. GAGs have the potential to modulate chemokine heterodimer formation and function, receptor binding and enhance stability [29,30,31]. GAG binding has been identified as essential for regulating chemotaxis in vivo [12], and could, therefore, be an aspect of chemokine biology to be targeted to modulate function. However, the system is intricate and complex, with the diversity of GAGs (which vary greatly in length, composition and sulphation pattern as shown in Figure 2), the oligomerisation state of the chemokine and the tissue microenvironment all affecting the chemokine-GAG interactions, and increasing the challenge of targeting this aspect of chemokine biology [32,33]. The presence/composition of other molecules beside GAGs also influences binding, for example, studies have shown that sialic acid and mannose-containing glycans are responsible (in addition to GAGs) for the binding of CCL5 to both CCR5+ and CCR5− cells [34]. Furthermore, data are beginning to show that chemokine residues that are involved in receptor interactions are also involved in GAG binding, suggesting GAG-bound chemokines may be unable to bind their receptors [27,29,35,36]. The affinity of the chemokine for different GAGs also changes depending upon whether the chemokine is in the monomer/dimer state, with dimers generally being the higher affinity GAG ligands [37,38,39]. The ratio of bound to free chemokine is therefore fine-tuned to modulate cellular recruitment.

The highly sulphated and acidic GAGs bind to basic residues within chemokines through electrostatic and H-bonding interactions. This usually involves residues such as arginine, lysine or histidine, which typically form the BBXB or (B)BXX(X/B)BXXB(B) peptide signature, where B is a basic amino acid residue and X a non-conserved amino acid, present in virtually all chemokines. Earlier studies revealed BBXB or (B)BXX(X/B)BXXB(B) as common heparin binding sequences for several chemokines, however, with the characterisation of more GAG-binding regions, it is suggested that GAG-binding motifs can be defined as sequential distant residues that form an optimal binding surface due to spatial orientation in the folded state [40]. This binding regulates the steepness and duration of chemokine gradients, which in turn regulates leukocyte adhesion and infiltration [41,42]. GAG binding has been identified as essential for the induction of chemotaxis, as chemokine mutants that bind receptor but not GAGs have impaired ability to recruit immune cells in vivo [12]. GAG binding could, therefore, be an aspect of chemokine biology to be targeted to modulate function.

### Common GAGs: Heparan Sulphate and Heparin

Heparan Sulphate (HS) is an anionic GAG component of the glycocalyx, and the most abundant GAG on the surface of endothelial cells [43]. HS is initially synthesised as a repeating disaccharide composed of the monomeric units *N*-acetyl-glucosamine (GlcNAc) and glucuronic acid. These units may or may not then be modified by a series of biosynthetic reactions within the Golgi. These give rise to *N*-, 6-*O*, or (albeit rarely) 3-*O*-sulphation of the glucosamine (GlcNS), as well as epimerisation and subsequent 2-*O*-sulphation of the glucuronic acid. The family of enzymes responsible for these modifications includes *N*-deacetylase/*N*-sulphotranferases (NDSTs 1/2/3/4), 2-*O*-sulphotransferases (HS2ST), 6-*O*-sulphotransferases (HS6ST), and 3-*O*-sulphotransferases (HS3ST) [44,45]. Mature HS can also be modified on the cell surface glycocalyx by specific sulphatases (SULF1 and SULF2). Additionally, heparanase, an endo-glycosidase, can cleave the HS polymer releasing smaller fragments from the HS proteoglycan complex.

HS serves homeostatic functions, including maintenance of the endothelial barrier permeability and the activation of antithrombin III. During disease or stress, HS can present inflammatory molecules such as chemokines to leukocytes, facilitating selectin-mediated rolling along the endothelial surface, potentially leading to increased integrin adhesion, intravascular arrest and diapedesis [46] (Figure 1).

In the short term, inflammation such as ischaemia-reperfusion injury can induce the shedding of some HS proteoglycans from the endothelial cell surface, which can then bind and sequester chemokines in the blood and reduce leukocyte migration [47,48,49]. Upon regeneration of the glycocalyx, upregulation of the expression of NDST enzymes increases the extent of *N*-sulphation, which in turn enhances the potential of the endothelium to bind and present pro-inflammatory chemokines [50]. This highlights the flexibility and varied regulation of endothelial GAGs and their ability to modulate chemokine binding and subsequent leukocyte migration.

Heparin, a soluble GAG produced by mast cells [51], has essentially the same backbone structure as HS but a different (more uniform) sulphation pattern [52]. Due to heparin’s uniform sulphation pattern, and the commercial availability of size-fractionated oligosaccharides of many different sizes, heparin is commonly used for structure—function and chemokine-GAG interaction studies.

## 4. Post-Translational Modification of Chemokines

The regulation of chemokines through post-translational modification can affect both receptor and GAG binding, and impact upon chemokine function and biological activity [53]. Many forms of modification can occur, such as cleavages by matrix metalloproteinases and other enzymes, as well as modifications of individual residues by citrullination or nitration [54,55,56,57].

The heterogeneous nature of post-translational modifications emphasises the need for better understanding, with some modifications enhancing or abrogating function, and others preventing detection using conventional methods [58,59]. This review article will focus on nitration, which occurs naturally during any situation that involves oxidative stress, such as myocardial infarction or organ transplantation.

## 5. Nitration of Chemokines

The reactive nitrogen species (RNS) peroxynitrite (ONOO^−^) is formed from the reaction between nitric oxide (NO) with the superoxide anion (O_2_^−^) [60,61]. ONOO^−^ has a very short half-life of around 10 ms at physiological pH, and can affect molecules within a 20 µm range of its production [62]. Effects of ONOO^−^ include protein nitration, lipid peroxidation, DNA strand breakage and the inhibition of cell signalling and metabolism [63].

NO is produced by nitric oxide synthase enzymes present in many cell types and in all tissues [64,65,66]. O_2_^−^ is produced by a range of enzymes present in many cell types, including nicotinamide adenine dinucleotide phosphate (NADPH) oxidase within the mitochondria [67,68,69]. Production of both NO [70] and O_2_^−^ [71,72] increases during inflammation and strategies to reduce production are protective in pre-clincial models of injury [73,74,75] and in human disease [76].

**Figure 3:**
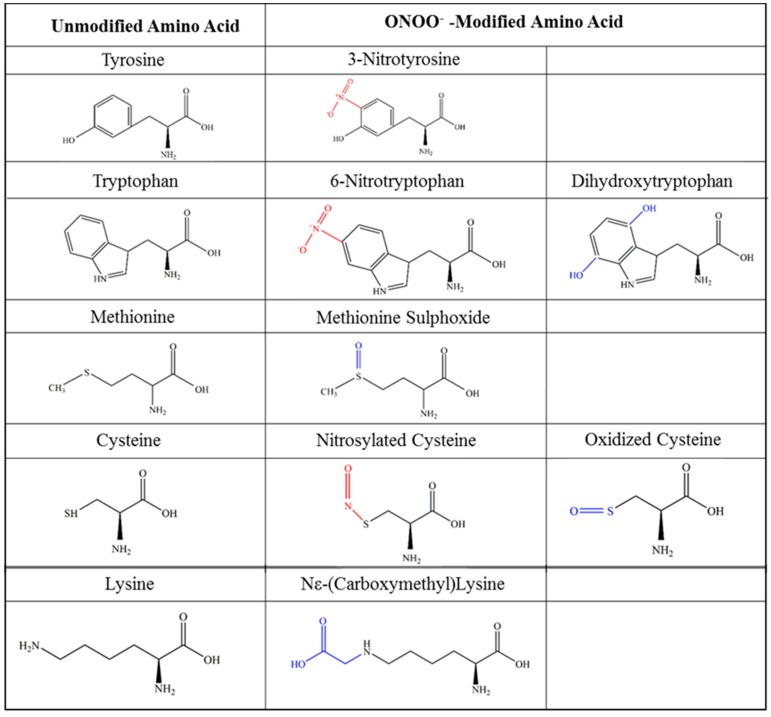
Some examples of amino acid modifications by peroxynitrite (ONOO^−^). Modifications involving oxidation are shown in blue, and modifications involving nitration are shown in red.

ONOO^−^ nitrates tyrosine residues to form 3-nitrotyrosine (3-NT), and also modifies tryptophan, cysteine, methionine, lysine and histidine, examples of which are shown in Figure 3 [77,78]. ONOO^−^ has been implicated in the pathology of many diseases [79], including myocardial reperfusion injury [80], cardiac allograft rejection [81], Fabry disease [82] and kidney diseases including acute tubular necrosis and diabetic nephropathy [83]. An increase in 3-NT was also detected in plasma and synovial fluid in osteoarthritis patients [84], in plasma from patients with interstitial lung disease [85] and type II diabetes mellitus [86].

One way that nitration could be affecting disease progression is through its effect on chemokines and leukocyte recruitment. Chemokine nitration usually results in a decrease in function [59] but for some proteins nitration can enhance function [87].

### 5.1. Effects of Nitration: Detection of Chemokines

Studies have shown that nitration may alter the ability of antibodies to detect proteins, presumably due to epitope modification by the addition of the NO_2_ groups. This has been shown for nitrated CCL2 and CXCL12 [54,88]. This may limit the biological relevance of measuring chemokine concentrations as disease biomarkers if only unmodified chemokine is detected. The amount of unmodified chemokine may be a less informative indicator of disease activity than the ratio of modified to unmodified chemokine.

### 5.2. Effects of Nitration: Chemotaxis

Nitration affects the chemotactic function of several chemokines but the biological significance of this is not fully understood. Incubation of chemokine with ONOO^−^ inhibits monocyte chemotaxis in response to CCL2 and eosinophil chemotaxis in response to CCL5 [89]. Another study found that CCL2 nitrated by intratumoural RNS was unable to induce CD8+ T cell recruitment to the tumour, but could still induce some recruitment of myeloid cells at high concentrations [88]. Nitration of tyrosine 7 in CXCL12 rendered the chemokine unable to induce lymphocyte chemotaxis both in vitro and in vivo [90]. Nitration could therefore be a negative regulator of inflammation; reducing the chemotactic functions of chemokines and thereby reducing leukocyte infiltration.

### 5.3. Effects of Nitration: Receptor Binding

The effect that nitration has on the ability of a chemokine to bind/signal through its receptor(s) is complex. Nitrated CCL2 was shown to have a reduced affinity for its receptor CCR2, which may explain its failure to induce chemotaxis of CD8+ T cells (as these cells express low levels of the CCR2 receptor), but retained ability to induce migration of myeloid cells (which express very high levels of CCR2) [88]. Nitration of CXCL12 does not affect its ability to bind the CXCR4 receptor, but does impair its ability to signal through this receptor [90]. In cases where nitration reduces receptor activation capacity, this could influence the receptor signaling bias mentioned previously, and increase the specificity of signaling in situations where many chemokines can bind to the same receptor.

To date, all research on nitration in chemokine biology appears to focus upon nitration of the chemokines themselves. The effect that nitration of the chemokine receptors may have is unknown. The Y188A CXCR1 mutant displayed a decreased affinity for CXCL8 compared with the wild type receptor, indicating the importance of this tyrosine residue in receptor-ligand interactions. As tyrosine is a potential target for nitration by ONOO^−^, nitration of CXCR1 as well as CXCL8 could affect receptor-ligand interactions [91].

### 5.4. Effects of Nitration: GAG Binding

Whether or not nitration affects GAG-binding depends upon the chemokine in question. For example, nitrated CXCL12 binds GAGs with a similar affinity as wild type CXCL12 [90], but nitrated CCL2 has been shown to have reduced ability to bind both heparin and heparan sulphate when compared to wild type CCL2 [92].

It is worth noting that soluble/immobilized chemokines can initiate different downstream pathways affecting cell migration, as is the case of the CCR7-CCL19/CCL21 axis. This means that in cases where nitration affects GAG binding (i.e., ability of the chemokine to be immobilized), this can in turn affect receptor signaling and therefore regulation of receptor binding, GAG binding and post-translational modifications are all likely to be linked and influence each other [93].

## 6. GAGs, Nitration and CXCL8 Function

CXCL8 is a potent neutrophil chemoattractant protein released by many cell types in response to a wide range of stimuli including cytokines, microbial products and hypoxia [94,95]. CXCL8 has also been shown to act on other cell types such as lymphocytes and fibroblasts, and is known to promote angiogenesis [96] and leukocyte degranulation. CXCL8 is therefore implicated in both acute and chronic inflammation [97]. Its modulation could influence the pathology of a wide range of diseases and at multiple disease stages [98].

### 6.1. Targeting CXCL8-GAG Interactions

**Figure 4:**
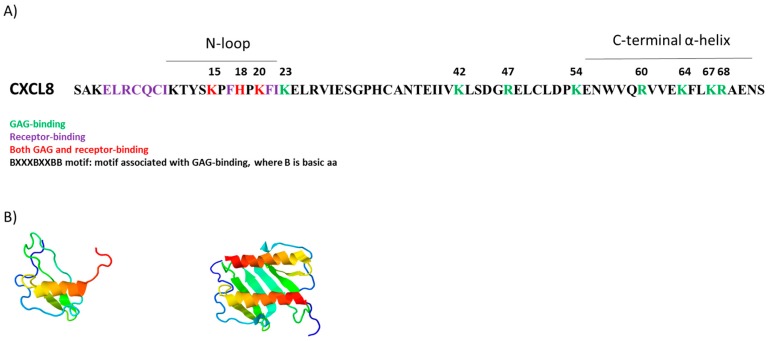
CXCL8 sequence and structure. (**A**) Diagrammatic representation of CXCL8 (72 amino acids long), showing the amino acid sequence. Purple: Receptor-binding residues. Green: GAG-binding residues. Red: residues implicated in both GAG and receptor binding; (**B**) CXCL8 in monomeric form (1KL, PDB) on the left, and dimeric form on the right (1CXCL8, PDB).

Studies have shown that while the CXCL8 monomer is the higher affinity receptor ligand, the CXCL8 dimer (which is the higher affinity GAG ligand) is far less competent at CXCR1 receptor activation (although quite active for CXCR2 [99]). This suggests that CXCL8, when GAG-bound, cannot access the receptor [36,100,101]. The C-terminal alpha helix of CXCL8, in addition to some basic residues located within the N-loop, is critical for GAG binding [102,103] due to its positive electrostatic charge. This binding is mediated by basic amino acids (Arg, Lys, His) core residues and by other secondary residues across its sequence (as shown in Figure 4) [41,104]. Targeted substitution of these basic residues for alanine residues reduced in vivo neutrophil recruitment to the peritoneum [8,32], but increased recruitment to the lungs [32,105]. These different recruitment patterns of neutrophils in response to CXCL8 in the mouse peritoneum compared to lung could be attributed to differences in chemokine gradients caused by different GAG structures and compositions between these tissues, and by differences in binding kinetics or diffusion rates, adding further complexity to this topic [32].

### 6.2. Competitive Displacement of Chemokines

The administration of a GAG, usually heparin, is a method that has been employed in pre-clinical models to modulate inflammation, and is thought to act through disruption of pre-formed chemokine gradients present on cell surface GAGs. Heparin in various forms inhibits leukocyte recruitment to mouse models of arthritis, traumatic brain injury and lipopolysaccharide (LPS) treatment [106,107,108], although its effectiveness depends upon the dose given and the duration of inflammation [109]. These studies show potential role of GAG mimetics on chemokine-mediated immunomodulation when administered, either local or systemically, however it should be noted that administered heparin is likely to interact with all cytokines due to its highly negative charge, and a more chemokine-specific gradient disruption method could be more beneficial.

Chemokine-GAG interactions also play an essential role in the antiviral immune response. Viruses can evade the chemokine-mediated immune response by expression of viral chemokine binding proteins (vCKBP), which interfere with the GAG binding, GPCR-binding, or both, thus modulating chemokine-mediated migration of leukocytes to the site of infection or tissue damage in vitro and in vivo [110].

### 6.3. Mutants with Altered GAG Binding

Substitution of basic residues for alanine residues in the GAG binding domain generates a non-GAG binding mutant. These mutant chemokines bind their cognate receptors normally and competitively inhibit binding of their wild type counterparts. Occupation of chemokine receptors by non-GAG binding chemokine variants prevents migration along a gradient and therefore inhibits chemotaxis, as has been shown with CCL5, CCL7 and CXCL12 amongst others [111,112]. Studies have shown that CXCL8 mutants with reduced GAG-binding abilities induced lower recruitment of neutrophils than wild type CXCL8 in the peritoneum but not the lung in vivo [32,105]. This work could be developed in order to create a non-GAG binding CXCL8 mutant with further impaired recruitment capabilities, although clearly biological activity effects in different tissues would need to be fully characterized. Studies conducted on CXCL11, however, showed that a mutant with reduced GAG binding in vitro could still induce cell migration in vivo, highlighting the need for each chemokine to be studied individually [113].

A variant of CXCL8 which has no ability to bind GPCRs but with increased GAG binding affinity inhibits trans-endothelial migration of neutrophils by displacing CXCL8 from the surface of endothelial cells [114]. A similar study by our group showed that a non-GPCR binding, increased-GAG binding CXCL12 variant showed a reduction in cell migration [115]. A CCL2 mutant with increased GAG binding was shown to displace multiple chemokines which could overcome the issues of redundancy [116], however high concentrations of chemokine may be required to occupy binding sites on all GAGs [43,117]. This approach represents another potential method of regulating chemokine function.

### 6.4. Using Peptides to Block Chemokine-GAG Binding

In addition to whole chemokine mutants, small peptide fragments of chemokines, for example, a CXCL9 C-terminal peptide was successfully able to compete with CXCL8, CXCL11 and CCL2 for binding to heparin, HS or other GAGs [118]. This illustrates the therapeutic potential of peptides to inhibit chemokine function by disrupting the interaction between chemokines and GAGs. In addition, these short chemokine fragments might occur naturally, due to cleavage by proteases such as matrix metalloproteinases (MMPs). Unpublished data from our group suggests that both a synthesised wild type (KENWVQRVVEKFLKRAENS) and mutant E70K CXCL8 peptide (KENWVQRVVEKFLKRAKNS) can successfully inhibit the action of the full length wild type protein, and thereby reduce adhesion of leukocytes to an endothelial cell monolayer under physiological flow conditions.

### 6.5. Nitration and CXCL8 Function

Neutrophils recruited by CXCL8 produce NO and reactive species generating ONOO^−^. Therefore nitration of CXCL8 is likely to occur at sites of inflammation. This could be a mechanism by which neutrophils limit further chemo-attraction to prevent tissue injury [119]. Unpublished data from our group suggests that nitration significantly reduces the ability of CXCL8 to induce neutrophil chemotaxis in vitro.

How nitration may affect the function of CXCL8 is as yet undetermined. Y13 is a residue in the *N*-loop that is known to be important for receptor signaling and a target for ONOO^−^. Nitration alters the p*K*_a_ making tyrosine residues more acidic, increases the mass of the protein by 45 Da per residue nitrated [54], and is also likely to cause some steric hindrance through increasing the surface area of tyrosine’s phenolic ring [120]. The nitration of tyrosine also affects its hydrophobicity, although there are conflicting reports in the literature as to whether this makes the residue more hydrophilic [70] or hydrophobic [120]. It is possible that the hydrophobicity of tyrosine is important in the function of CXCL8 in particular, as a Y13L mutant (which maintains hydrophobicity) showed similar if not slightly increased activity when compared to the wild type [121], but Y13E (hydrophilic) and Y13T (neutral) mutants both showed a decrease in receptor affinity [122]. As the core and secondary GAG-binding residues of CXCL8 described previously include histidines and lysines, which are potential targets of ONOO^−^, it is likely that modification of CXCL8 by ONOO^−^ could also affect its GAG binding properties [123].

Tyrosine has also been shown to be an important residue within the receptor CXCR1, as a Y188A mutant version showed decreased affinity for CXCL8 in comparison to the wild type receptor [91]. Therefore nitration of the receptors as well as the ligands (particularly tyrosine residues) could affect chemokine-mediated signal transduction and leukocyte chemotaxis. It is possible that the location and function of the aforementioned residues within any given chemokine (and/or receptor) will determine the specific effects of nitration on each one in turn, highlighting the need for further study.

## 7. Future Research Directions

Factors such as chemokine-GAG binding and post-translational protein modification are increasingly recognised as important determinants of chemokine function in vivo. How these factors affect chemokine function is only starting to emerge and the challenge is now to understand their effects at a whole organ/organism level during both normal tissue homeostasis and in disease. This is not only of biological interest but it may identify new treatment targets.

In this review we have discussed the importance of chemokine-GAG interactions and how this could be modified by soluble GAGs, mutant chemokines or peptide fragments. There is increasing evidence that this can be done in vitro and in pre-clinical disease models. However, we still do not know what the effect of disrupting chemokine gradients in injured tissues would be nor how this could be applied in the clinic. These are all important areas of future research.

The capacity to mount an effective inflammatory response is paramount. However, to maintain tissue integrity, this response has to be regulated. If we understand the natural mechanisms employed to control inflammation we may be able to exploit this to modify disease. One example discussed in this review is the nitration of chemokines, with resultant loss of activity. Currently, the best methods for detecting chemokine nitration involve NMR analysis or Nano-HPLC, however the development of antibodies specific for nitrated chemokines would better facilitate their study; something our group is currently investigating for nitrated CXCL8. This and similar chemokine modifications could be biological ‘off switches’, limiting unopposed leukocyte accumulation and tissue damage. Studies are beginning to find links between these different regulatory aspects of chemokine biology, and clearly further study is required to discover how post-translational modifications may affect GAG and GPCR binding in order to contribute to a more complete understanding of the biology of chemokine regulation.

## Abbreviations

ACKR	Atypical chemokine receptor
ECM	Extracellular matrix
GAG	Glycosaminoglycan
Gal	Galactose
GalNAc	*N*-acetyl-galactosamine
GlcA	Glucuronic acid
GlcNAc	*N*-acetyl-glucosamine
GlcNS	Glucosamine
GPCR	G-protein coupled receptor
HS	Heparan sulphate
HS2ST	2-*O*-sulphotransferases
HS6ST	6-*O*-sulphotransferases
HS3ST	3-*O*-sulphotransferases
IdoA	Iduronic acid
LPS	Lipopolysaccharide
MMPs	Matrix metalloproteinases
NADPH	Nicotinamide adenine dinucleotide phosphate
NDSTs+	*N*-deacetylase/*N*-sulphotranferases
NO	Nitric oxide
O_2_^−^	Superoxide anion
ONOO^−^	Peroxynitrite
PTM	Post-translational modifications
RNS	Reactive nitrogen species
SULF1/2	Sulphatases
vCKBP	Viral chemokine binding proteins
3-NT	3-Nitrotyrosine
